# The association between circadian syndrome and chronic kidney disease in an aging population: a 4-year follow-up study

**DOI:** 10.3389/fendo.2024.1338110

**Published:** 2024-04-26

**Authors:** Yang Xiong, Qian Zhong, Yangchang Zhang, Zhihong Liu, Xianding Wang

**Affiliations:** ^1^ Department of Urology and Andrology Laboratory, West China Hospital, Sichuan University, Chengdu, Sichuan, China; ^2^ Department of Endocrinology, West China Hospital, Sichuan University, Chengdu, Sichuan, China; ^3^ Department of Public Health, Capital Medical University, Beijing, China; ^4^ Kidney Transplant Center, Transplant Center, West China Hospital, Sichuan University, Chengdu, Sichuan, China

**Keywords:** aging, chronic kidney disease, circadian syndrome, CHARLS, Chinese

## Abstract

**Introduction:**

Circadian syndrome (CircS) is proposed as a novel risk cluster based on reduced sleep duration, abdominal obesity, depression, hypertension, dyslipidemia and hyperglycemia. However, the association between CircS and chronic kidney disease (CKD) remains unclear. To investigate the cross-sectional and longitudinal association between CircS and CKD, this study was performed.

**Methods:**

A national prospective cohort (China Health and Retirement Longitudinal Study, CHARLS) was used in this study. To define CKD, the estimated glomerular filtration rate (eGFR) was calculated based on the 2012 CKD-EPI creatinine-cystatin C equation. Participants with eGFR <60 mL.min^-1^/1.73/m^2^ were diagnosed with CKD. Multivariate binary logistic regression was used to assess the cross-sectional association between CircS and CKD. Subgroup and interactive analyses were performed to determine the interactive effects of covariates. In the sensitivity analysis, the obese population was excluded and another method for calculating the eGFR was used to verify the robustness of previous findings. In addition, participants without CKD at baseline were followed up for four years to investigate the longitudinal relationship between CircS and CKD.

**Results:**

A total of 6355 participants were included in this study. In the full model, CircS was positively associated with CKD (OR = 1.28, 95% CI = 1.04-1.59, *P* < 0.05). As per one increase of CircS components, there was a 1.11-fold (95% CI = 1.04-1.18, *P* < 0.05) risk of prevalent CKD in the full model. A significant interactive effect of hyperuricemia in the CircS-CKD association (*P* for interaction < 0.01) was observed. Sensitivity analyses excluding the obese population and using the 2009 CKD-EPI creatinine equation to diagnose CKD supported the positive correlation between CircS and CKD. In the 2011-2015 follow-up cohort, the CircS group had a 2.18-fold risk of incident CKD (95% CI = 1.33-3.58, *P* < 0.01) in the full model. The OR was 1.29 (95% CI = 1.10-1.51, *P* < 0.001) with per one increase of CircS components.

**Conclusion:**

CircS is a risk factor for CKD and may serve as a predictor of CKD for early identification and intervention.

## 1 Introduction

Throughout history, scholars and researchers have diligently investigated the correlation between the circadian clock system and both diseases and individuals’ physiological states (1). The term “circadian syndrome (CircS)” is employed to denote the phenomenon through which living organisms, spanning multiple hierarchical levels, from molecules and cells to organisms and populations, have undergone adaptive evolutionary processes resulting in the emergence of periodic fluctuations in their life activities as a response to diurnal oscillations in their environmental conditions (2). The concept of CircS is grounded in compelling evidence that several chronic disorders, including obesity, hypertension, dyslipidemia, type 2 diabetes, depression, sleep disorders, and nonalcoholic fatty liver disease, are strongly associated with circadian rhythms (3). CircS serves as the principal regulator influencing nearly every facet of human health and metabolism (1). Various physiological parameters within the human body, encompassing metrics such as pulse rate, body temperature, blood pressure, physical endurance, emotional states, cognitive function, metabolism, organ function, hormone secretion, immunity, and the cell cycle, exhibit periodic fluctuations synchronized with diurnal and nocturnal shifts (4–6).

The central pacemaker of the circadian clock resides within the suprachiasmatic nucleus (SCN) of the brain and synchronizes with external cues, primarily light (2). The central clock synchronizes peripheral clocks located in various regions of the brain and tissues throughout the body, utilizing both neuronal and humoral signaling pathways. While light holds dominance as the Zeitgeber for central clock entrainment, metabolic cues are likely to function as supplementary Zeitgebers for peripheral clocks, particularly those situated in the liver and kidneys (4, 7). It has been confirmed that the kidneys exhibit circadian rhythms, with functions such as urine volume, glomerular filtration rate (GFR), sodium excretion, renal plasma flow (RPF), urine protein excretion rate, urine electrolyte excretion rate, blood pressure, glucocorticoids, erythropoietin, endothelin, and others showing circadian variations (7–10). Chronic kidney disease (CKD) is an umbrella term for various kidney diseases characterized by irreversible structural and functional abnormalities lasting for several months (11). The prevailing criteria employed in the adult population for defining CKD encompass: (1) evidence of kidney impairment, frequently identified through an elevated urine albumin (or protein)-to-creatinine ratio (ACR); or (2) diminished kidney function, characterized by a GFR below 60 ml/min per 1.73 m^2^. GFR is considered the best determinant of kidney function (12). CKD’s main causes vary by region, with diabetes being a leading factor in white individuals, while glomerulonephritis plays a significant role in Asia, but microvasculature dysfunction is a common underlying mechanism (13).

Currently, there is increasing evidence suggesting that modern lifestyles (such as sleep deprivation, lack of physical activity, high-energy diets, and shift work) and the use of artificial lighting can disrupt circadian rhythms, leading to various health consequences and potentially being important factors in the development of CKD (3, 14). Daily rhythmicity is essential for optimal kidney function, as the kidney houses one of the most active peripheral clocks. Disruptions in circadian rhythms have been linked to the onset of CKD in both human studies and animal models (15). Studies by Knutson et al. (16) reported an association between poor sleep quality and lower estimated glomerular filtration rate (eGFR) and higher urine protein to creatinine ratio (PCR) in patients with mild to moderate CKD. A cross-sectional study involving Chinese participants also found an association between poor sleep quality and higher odds of being at high risk for CKD and proteinuria (17). Additionally, cardiovascular events such as arrhythmias and acute myocardial infarctions, as the leading causes of death in CKD patients, also exhibit circadian rhythm characteristics, with higher incidences occurring at night and in the early morning (18). Conversely, CKD can further exacerbate circadian rhythm disturbances. A study in Spain showed that CKD patients had a higher proportion of disrupted blood pressure circadian rhythms, and this condition increased the risk of entering end-stage renal disease (ESRD) (19). In addition, some scholars have found that hemodialysis patients experience circadian rhythm disruption in peripheral tissues, manifesting as diminished nocturnal sleep quality, increased daytime sleepiness, and alterations in circadian biomarkers, such as melatonin (20).

Therefore, CircS may be considered a new cluster of risk factors for chronic kidney disease. However, there is currently no research indicating the relationship between CircS and CKD. The purpose of this study is to use data from the China Health and Retirement Longitudinal Study (CHARLS) to address this question.

## 2 Materials and methods

### 2.1 Data sources and study populations

This study derived from CHARLS, which is an ongoing longitudinal survey in the Chinese aging population. Initiated in 2011, CHARLS used the probabilities proportional to size method to sample across China. The included participants were all invited to receive a follow-up every 2-3 years. To date, CHARLS has launched four waves of investigation in 2011, 2013, 2015 and 2018. However, only the 2011 and 2015 surveys collected blood samples. Thus, we used the two waves to construct the longitudinal cohort. A more detailed description of CHARLS can be found in the protocol of the CHARLS project or the official website (http://charls.pku.edu.cn/) (21). The ethical review board of Peking University meticulously examined and subsequently sanctioned this study (IRB 00001052-11014; Beijing, China). Prior to their participation in this study, written and oral informed consent was obtained from all participants.

**Figure 1:**
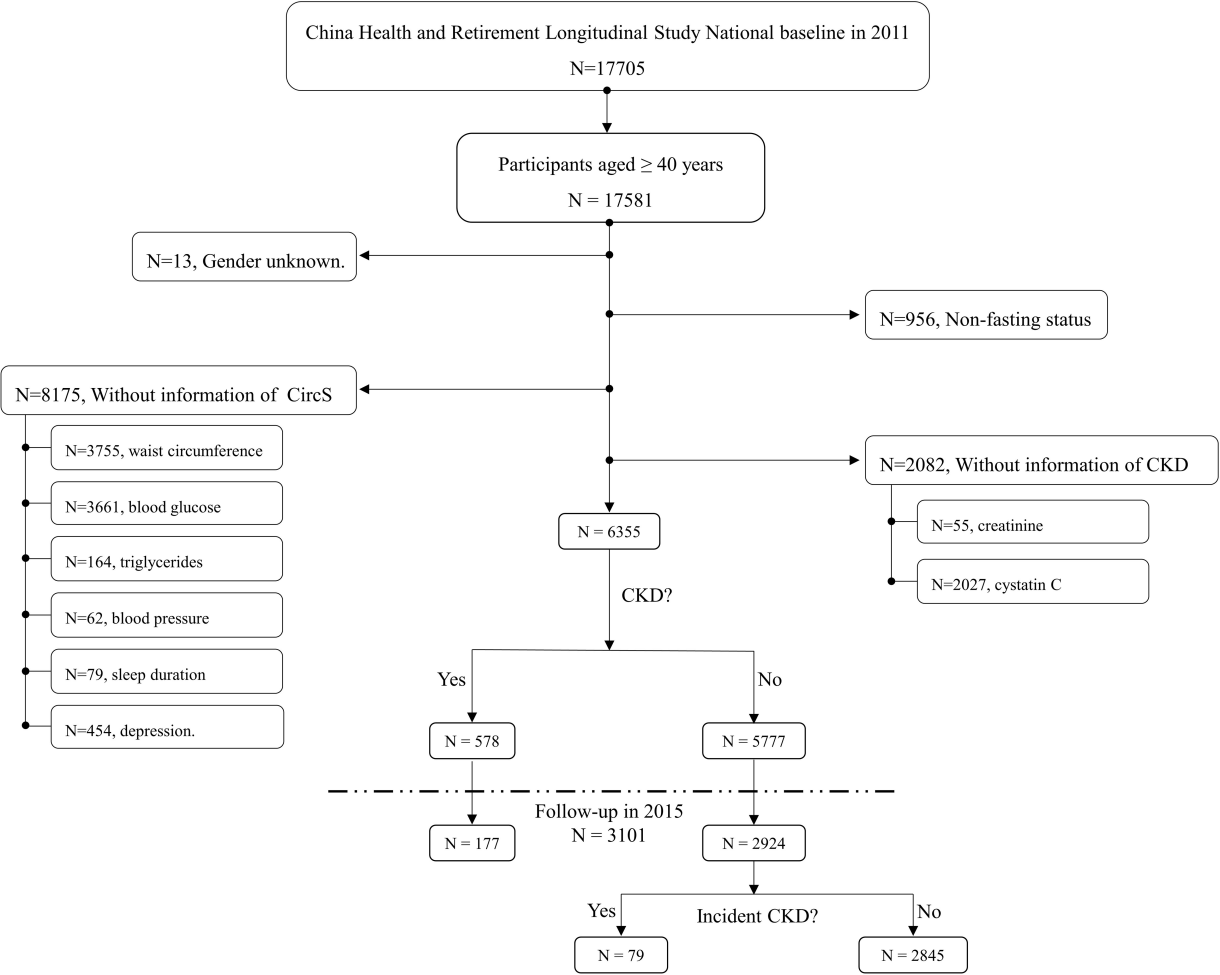
Overview of the study design and analysis strategy. In CHARLS, we first investigated the cross-sectional association between CircS and prevalent CKD in 2011 and the longitudinal association was explored in a four-year follow-up survey from 2011-2015.

In the baseline survey, CHARLS sampled 17705 participants from 28 provinces. As shown in **Figure 1**, participants aged <40 years, under non-fasting status and having incomplete information to diagnose CircS and CKD were excluded. Finally, 6355 participants were included in the cross-sectional analysis. Among them, 3101 participants were followed up to 2015 and then included in the longitudinal analysis.

### 2.2 Measurements of CircS and CKD

Derived from metabolic syndrome, the concept of CircS is built based on seven components: reduced sleep duration, depression, central obesity, elevated triglycerides, decreased HDL, hypertension and hyperglycemia. Participants with at least four components simultaneously are defined as having CircS (3). The duration of sleep was evaluated through individual self-reporting, and a sleep duration of less than six hours was classified as reduced sleep duration. Depression was assessed using the Center for Epidemiological Studies Depression Scale-10 (CESD-10) questionnaire (22). Participants with scores ≥10 were diagnosed with depression. Central obesity was defined as waist circumference ≥85 cm for males and ≥80 cm for females.

To determine the concentrations of triglycerides, HDL and blood glucose in fasting status, all the participants were asked to fast overnight. The next morning, venous blood was collected by medical staff and centrifuged to separate the plasma. Participants under non-fasting status were not excluded in this step; however, they were labeled and subsequently excluded from the formal analysis. The collected plasma was immediately stored at −20°C and then transported to −80°C for long-term preservation. All determinations were performed at the Capital Medical University laboratory using enzymatic colorimetric tests. According to one previous study, elevated triglyceride was defined as triglycerides ≥150 mg dL^−1^ or drug treatment for elevated triglycerides (3). Reduced HDL was defined as HDL <40 mg dL^−1^ for males or <50 mg dL^−1^ for females or drug treatment for reduced HDL. Elevated fasting glucose was defined as ≥100 mg dL^−1^ or drug treatment of elevated glucose. To measure the blood pressure, the participants were asked to be relaxed and seated for a while. Blood pressure was assessed three times by experienced nurses using a HEM-7200 electronic monitor (Omron, Dalian). The average of three measurements was used as the final blood pressure. Hypertension was defined as systolic pressure ≥140 mmHg or diastolic pressure ≥90 mmHg or drug treatment of hypertension. All medication history was based on the patient’s self-report.

To define CKD, we first calculated the estimated glomerular filtration rate (eGFR) based on the 2012 CKD-EPI creatinine-cystatin C equation (23). Patients with eGFR <60 mL min^-1^/1.73/m^2^ were diagnosed with CKD (24).

### 2.3 Measurements of covariates

Some covariates, including demographic variables, lifestyle variables and blood biomarkers were collected in the baseline survey. Demographic variables included age (years), educational levels (literate and illiterate) and marital status (married with spouse/cohabitating versus divorced/separated/widowed). Here, illiterate refers to participants who received less than an elementary school education. Lifestyle factors comprised afternoon nap (yes or no), cigarette consumption (current, never or ex-smoker) and alcohol consumption (more than once a month, less than once a month, and never). Blood biomarkers consisted of low-density lipoprotein (LDL, mg/dL), total cholesterol (mg/dL), hyperuricemia, C-reactive protein (CRP, mg/L) and hemoglobin (g/dL). Hyperuricemia was defined as blood uric acid >420 μmol/L for males and >360 μmol/L for females (25).

### 2.4 Statistic analysis

The baseline characteristics of the participants were summarized according to the status of CircS (normal group versus CircS group). Continuous variables with normal distribution were displayed as mean ± standard deviation and those with non-normal distribution were displayed as median (25%-75% quartiles). Categorical variables were showed as proportions (%). The differences between the two groups were tested using t-test, Wilcoxon test or Chi-square test according to the data types.

To evaluate the cross-sectional association between CircS and CKD, binary logistic regression was used. A set of models was constructed by adjusting different covariates: Model 1 – crude model; Model 2 - adjusting for age, gender, marital status and educational levels; Model 3 – further adjusting for cigarette and alcohol consumption and afternoon nap; Model 4 – adjusting for blood biomarkers, including LDL, total cholesterol, hyperuricemia, CRP and hemoglobin. The participants were followed up to 2015 to investigate the longitudinal association between CircS and CKD.

In addition, some sensitivity analyses were performed. First, we used the number of CircS components as a continuous variable to verify the cross-sectional and longitudinal associations. Second, subgroup and interactive analyses were performed to explore the potential interactive effects of covariates. Third, we excluded the obese population (body mass index ≥28.0 Kg/m^2^) to verify the association between CircS and CKD in the non-obese population. Finally, given that there is no consensus in the equation for estimating eGFR, we also used the 2009 CKD-EPI creatinine equation to calculate the eGFR as a sensitivity analysis (23).

All analyses in this study were performed using R 4.0.2 software (R Foundation for Statistical Computing, Vienna, Austria). A *P*-value < 0.05 (two-sided) indicates statistical significance.

## 3 Results

### 3.1 Characteristics of participants attending the 2011 CHARLS baseline survey

As shown in **Figure 1**, 6355 participants were included in the baseline survey. Among them, 2506 participants were diagnosed with CircS, and 3849 were not (**Table 1**). The CircS group tended to be older, female, divorced/separated/widowed, illiterate, have less cigarette and alcohol consumption and have higher LDL, total cholesterol, CRP, uric acid and hemoglobin (all *P* < 0.05).

**Table 1:** Baseline characteristics of participants attending the CHARLS survey in 2011.

Characteristics	Normal N = 3849	CircS N = 2506	Overall N = 6355	*P*
Age (years)	59.72 ± 10.09	61.22 ± 9.72	60.31 ± 9.97	<0.001
Gender				
Male	2091 (54.33%)	886 (35.36%)	2977 (46.85%)	<0.001
Female	1758 (45.67%)	1620 (64.64%)	3378 (53.15%)	
Marital status				
Married/cohabitating	3245 (84.31%)	2009 (80.17%)	5254 (82.68%)	<0.001
Others	604 (15.69%)	497 (19.83%)	1101 (17.32%)	
Educational levels				
Literate	2073 (53.86%)	1202 (47.96%)	3275 (51.53%)	<0.001
Illiterate	1776 (46.14%)	1304 (52.04%)	3080 (48.47%)	
Has afternoon nap	2107 (54.74%)	1404 (56.05%)	3511 (55.26%)	0.310
Cigarette consumption				
Current smoker	1369 (35.58%)	569 (22.71%)	1938 (30.50%)	<0.001
Non-smoker	2129 (55.33%)	1698 (67.76%)	3827 (60.23%)	
Ex-smoker	350 (9.10%)	239 (9.54%)	589 (9.27%)	
Alcohol consumption				
Drink more than once a month	1108 (28.79%)	445 (17.76%)	1553 (24.44%)	<0.001
Drink but less than once a month	297 (7.72%)	183 (7.30%)	480 (7.55%)	
None of these	2444 (63.50%)	1878 (74.94%)	4322 (68.01%)	
BMI (Kg/m^2^)				
<18.5	384 (10.03%)	69 (2.79%)	453 (7.19%)	<0.001
18.5-24.0	2380 (62.17%)	899 (36.35%)	3279 (52.04%)	
24.0-28.0	821 (21.45%)	998 (40.36%)	1819 (28.87%)	
≥28.0	243 (6.35%)	507 (20.50%)	750 (11.90%)	
LDL (mg/dL)	116.37 ± 32.35	119.98 ± 39.04	117.79 ± 35.18	<0.001
Total cholesterol (mg/dL)	189.89 ± 36.29	201.00 ± 41.62	194.27 ± 38.86	<0.001
Hyperuricemia	147 (3.82%)	208 (8.30%)	355 (5.59%)	<0.001
C-reactive protein (mg/L)	0.86 (0.49-1.82)	1.35 (0.71-2.78)	1.06 (0.56-2.19)	<0.001
Hemoglobin (g/dL)	14.29 ± 2.18	14.47 ± 2.27	14.36 ± 2.22	0.001
Depression	1113 (28.92%)	1378 (54.99%)	2491 (39.20%)	<0.001
Sleep duration (<6 hours)	803 (20.86%)	1115 (44.49%)	1918 (30.18%)	<0.001
Hypertension	1447 (37.59%)	1930 (77.02%)	3377 (53.14%)	<0.001
Systolic pressure (mmHg)	126.48 ± 24.05	139.94 ± 26.49	131.76 ± 25.88	<0.001
Diastolic pressure (mmHg)	73.22 ± 11.47	79.35 ± 11.99	75.63 ± 12.05	<0.001
Abdominal obesity	1566 (40.69%)	2145 (85.59%)	3711 (58.39%)	<0.001
Reduced serum HDL	765 (19.88%)	1844 (73.58%)	2609 (41.05%)	<0.001
Elevated serum triglycerides	330 (8.57%)	1498 (59.78%)	1828 (28.76%)	<0.001
eGFR (ml.min^−1^/1.73 m^2^)	84.53 ± 17.10	83.70 ± 19.01	84.20 ± 17.88	0.070
CKD	305 (7.92%)	273 (10.89%)	578 (9.10%)	<0.001

Data are presented as mean ± standard error (SD) for continuous measures with normal distribution and median (25–75% quantiles) with non-normal distribution, and n (%) for categorical measures. The differences of covariates across groups were tested using t test or Wilcoxon test for continuous variables and Chi-square test for categorical data. The others group in marital status refers to the divorced/separated/widowed. BMI, body mass index; CHARLS, China Health and Retirement Longitudinal Study; LDL, low density lipoprotein; HDL, high density lipoprotein; eGFR, estimated glomerular filtration rate; CKD, chronic kidney disease.

### 3.2 The cross-sectional association between CircS and prevalent CKD

The CircS group had a significantly higher risk of prevalent CKD (**Table 2**). In the crude model, the CircS group had a 1.42-fold risk of prevalent CKD (95% CI = 1.20-1.69, *P* < 0.001). After adjusting for different covariates, the ORs were 1.39 (95% CI = 1.14-1.69, *P* = 0.001), 1.38 (95% CI = 1.13-1.68, *P* = 0.002) and 1.28 (95% CI = 1.04-1.59, *P* < 0.05) in model 2, model 3 and the full model (model 4), respectively. As a continuous variable, the ORs were 1.16 (95% CI = 1.10-1.22, *P* < 0.001), 1.14 (95% CI = 1.07-1.21, *P* < 0.001), 1.14 (95% CI = 1.07-1.21, *P* < 0.001), and 1.11 (95% CI = 1.04-1.18, *P* < 0.05) in the crude model (model 1), model 2, model 3 and the full model (model 4), respectively.

**Table 2:** The cross-sectional association between CircS and prevalent CKD.

Models	Per one of CircS component (continuous)		CircS (yes versus no)	
	OR (95% CI)	*P*	OR (95% CI)	*P*
Model 1	1.16 (1.10-1.22)	<0.001	1.42 (1.20-1.69)	<0.001
Model 2	1.14 (1.07-1.21)	<0.001	1.39 (1.14-1.69)	0.001
Model 3	1.14 (1.07-1.21)	<0.001	1.38 (1.13-1.68)	0.002
Model 4	1.11 (1.04-1.18)	<0.001	1.28 (1.04-1.59)	0.020

Model 1 – crude model; Model 2 - adjusting for age, gender, marital status and educational levels; Model 3 – further adjusting for cigarette and alcohol consumption and afternoon nap; Model 4 – adjusting for blood biomarkers including LDL, total cholesterol, hyperuricemia, CRP and hemoglobin.

### 3.3 Association between CircS and prevalent CKD in subgroup and interactive analyses

**Figure 2:**
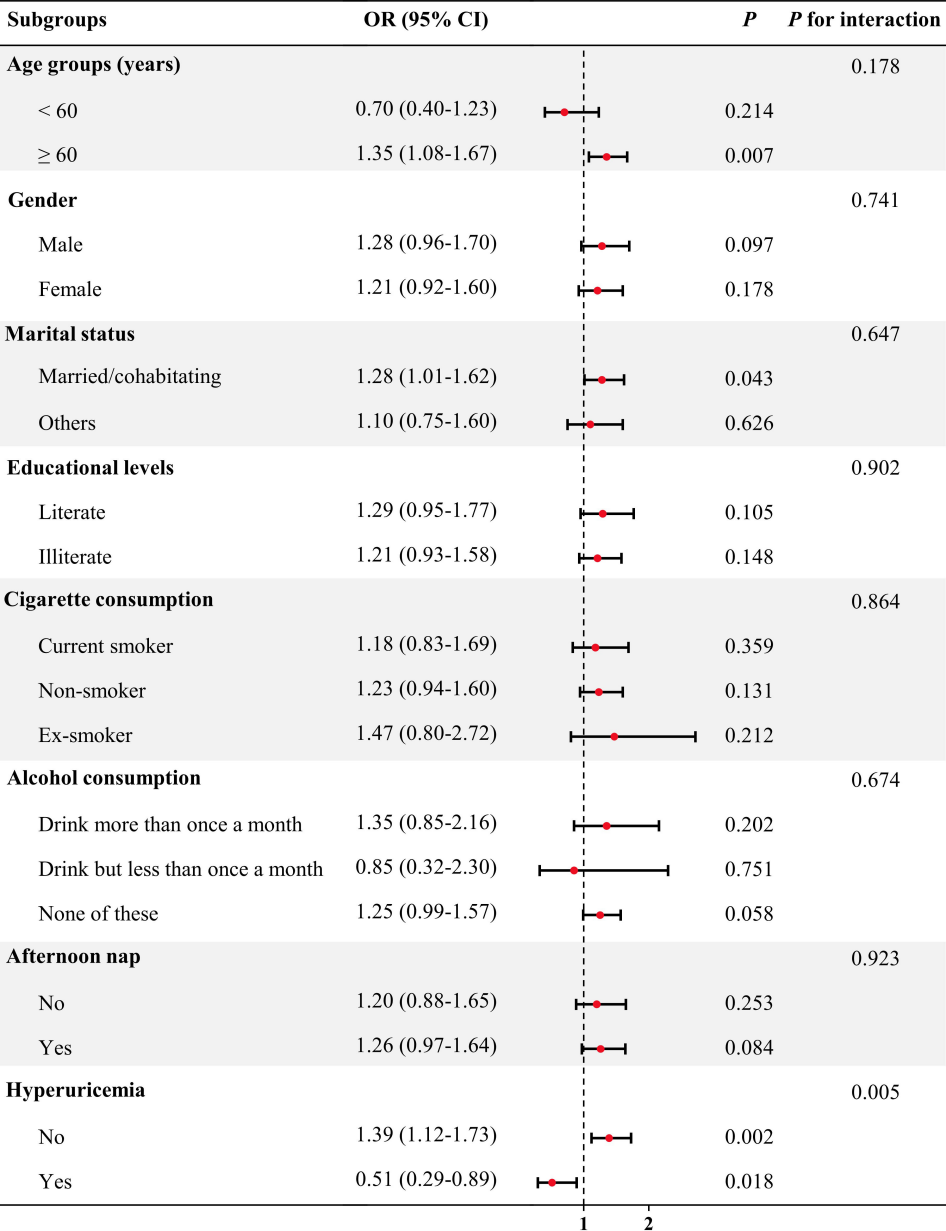
Association between CircS and prevalent CKD in subgroup and interactive analyses. In the multivariable logistic regression models, covariates were adjusted as model 4 in previous analyses except for subgroup variables.

In the subgroup analysis (**Figure 2**), an increased risk of prevalent CKD was observed in participants aged ≥60 years (OR = 1.35, 95% CI = 1.08-1.67, *P* = 0.007), being married/cohabitating (OR = 1.28, 95% CI = 1.01-1.62, *P* = 0.043) and not having hyperuricemia (OR = 1.39, 95% CI = 1.12-1.73, *P* = 0.002). Notably, the CircS group had a decreased risk of prevalent CKD for participants with hyperuricemia (OR = 0.51, 95% CI = 0.29-0.89, *P* = 0.018). there was a significant interactive effect of hyperuricemia in the CircS-CKD association (*P* for interaction < 0.01).

### 3.4 Association between CircS and prevalent CKD in the non-obese population

To assess the association between CircS and prevalent CKD in the non-obese population, an additional sensitivity analysis was conducted in which participants with obesity (body mass index ≥28.0 Kg/m^2^) were excluded (**Table 3**). The CircS group had a significantly higher risk of prevalent CKD in the crude model (OR = 1.54, 95% CI = 1.28-1.85, *P* < 0.001) and the full model (OR = 1.39, 95% CI = 1.11-1.74, *P* < 0.01). As a continuous variable, it was found that as per one increase of CircS components, the ORs were 1.20 (95% CI = 1.13-1.26, *P* < 0.001) in the crude model and 1.14 (95% CI = 1.06-1.23, *P* < 0.001) in the full model. In the non-obese population, CircS was also positively associated with prevalent CKD.

**Table 3:** The cross-sectional association between CircS and prevalent CKD in the non-obese population.

Models	Per one of CircS component (continuous)		CircS (yes versus no)	
	OR (95% CI)	*P*	OR (95% CI)	*P*
Model 1	1.20 (1.13-1.26)	<0.001	1.54 (1.28-1.85)	<0.001
Model 2	1.16 (1.09-1.24)	<0.001	1.45 (1.18-1.79)	<0.001
Model 3	1.16 (1.09-1.24)	<0.001	1.45 (1.18-1.79)	<0.001
Model 4	1.14 (1.06-1.23)	<0.001	1.39 (1.11-1.74)	0.004

Model 1 – crude model; Model 2 - adjusting for age, gender, marital status and educational levels; Model 3 – further adjusting for cigarette and alcohol consumption and afternoon nap; Model 4 – adjusting for blood biomarkers including LDL, total cholesterol, hyperuricemia, CRP and hemoglobin.

### 3.5 Association between CircS and prevalent CKD using the 2009 CKD-EPI creatinine equation

Given that there is no consensus in the equation for estimating eGFR, we also used the 2009 CKD-EPI creatinine equation to calculate the eGFR as a sensitivity analysis (**Table 4**). Using the new equation to calculate eGFR and further diagnose CKD, the CircS group still displayed a significantly higher risk of prevalent CKD in the crude model (OR = 1.71, 95% CI = 1.35-2.19, *P* < 0.001) and the full model (OR = 1.42, 95% CI = 1.07-1.89, *P* < 0.05). As a continuous variable, it was found that with per one increase of CircS components, the ORs were 1.22 (95% CI = 1.13-1.31, *P* < 0.001) in the crude model and 1.10 (95% CI = 1.01-1.21, *P* < 0.001) in the full model. The positive correlation between CircS and CKD was still valid.

**Table 4:** The cross-sectional association between CircS and prevalent CKD using 2009 CKD-EPI creatine equation.

Models	Per one of CircS component (continuous)		CircS (yes versus no)	
	OR (95% CI)	*P*	OR (95% CI)	*P*
Model 1	1.22 (1.13-1.31)	<0.001	1.71 (1.35-2.19)	<0.001
Model 2	1.19 (1.10-1.29)	<0.001	1.65 (1.27-2.14)	<0.001
Model 3	1.18 (1.09-1.28)	<0.001	1.61 (1.24-2.09)	<0.001
Model 4	1.10 (1.01-1.21)	<0.001	1.42 (1.07-1.89)	0.016

Model 1 – crude model; Model 2 - adjusting for age, gender, marital status and educational levels; Model 3 – further adjusting for cigarette and alcohol consumption and afternoon nap; Model 4 – adjusting for blood biomarkers including LDL, total cholesterol, hyperuricemia, CRP and hemoglobin.

### 3.6 The longitudinal association between CircS and incident CKD

To investigate the longitudinal association between CircS and incident CKD, a 2011-2015 follow-up cohort was constructed. The elderly population without CKD in 2011 were further followed up for four years to investigate the occurrence of incident CKD in 2015. As summarized in **Table 5**, the CircS group had a 2.36-fold risk of incident CKD (95% CI = 1.50-3.73, *P* < 0.001) in the crude model. After adjusting for different covariates, the ORs for incident CKD were 2.26 (95% CI = 1.41-3.62, *P* < 0.001), 2.28 (95% CI = 1.42-3.67, *P* < 0.001) and 2.18 (95% CI = 1.33-3.58, *P* < 0.01) in model 2, model 3 and the full model (model 4), respectively. As a continuous variable, the ORs were 1.31 (95% CI = 1.14-1.50, *P* < 0.001), 1.28 (95% CI = 1.11-1.48, *P* < 0.001), 1.29 (95% CI = 1.11-1.49, *P* < 0.001), and 1.29 (95% CI = 1.10-1.51, *P* < 0.001) in the crude model (model 1), model 2, model 3 and the full model (model 4), respectively.

**Table 5:** The longitudinal association between CircS and incident CKD.

Models	Per one of CircS component (continuous)		CircS (yes versus no)	
	OR (95% CI)	*P*	OR (95% CI)	*P*
Model 1	1.31 (1.14-1.50)	<0.001	2.36 (1.50-3.73)	<0.001
Model 2	1.28 (1.11-1.48)	<0.001	2.26 (1.41-3.62)	<0.001
Model 3	1.29 (1.11-1.49)	<0.001	2.28 (1.42-3.67)	<0.001
Model 4	1.29 (1.10-1.51)	<0.001	2.18 (1.33-3.58)	0.002

Model 1 – crude model; Model 2 - adjusting for age, gender, marital status and educational levels; Model 3 – further adjusting for cigarette and alcohol consumption and afternoon nap; Model 4 – adjusting for blood biomarkers including LDL, total cholesterol, hyperuricemia, CRP and hemoglobin.

## 4 Discussion

To the best of our knowledge, this is the first study to examine the longitudinal association between CircS and CKD among the elderly population aged over 40 years in Chinese communities using nationally representative data. In this population-based prospective cohort study, we observed a positive association between CircS and CKD in the cross-sectional analyses. After a 4-year follow-up period, newly developed CKD was significantly associated with CircS.

Previous research on CircS and CKD has primarily concentrated on specific behaviors, such as irregular or short-duration sleep, shift work, and exposure to artificial lighting (26). Fewer studies have utilized the concept of CircS to categorize individuals and investigate its relationship with health outcomes. Regarding the relationship between sleep duration and physical health issues, numerous studies have demonstrated that individuals who sleep for a short duration (< 6 hours per night) have an elevated risk of hypertension (RR 3.59, 95% CI 1.58-8.17) and CKD (RR 1.63, 95% CI 1.28-2.08) when compared to those who report sleeping 7 hours per night (15, 27, 28). A cohort study indicated a significant association between long-term night shift work and early kidney dysfunction in steelworkers (29). Another observational study found that a minor increase in albuminuria, which serves as an indicator of kidney damage, was linked to the disruption of circadian rhythms caused by shift work, rather than exposure to low levels of nephrotoxic chemicals (30). Concurrently, epidemiological investigations have revealed a higher prevalence of CKD among individuals with either excessively long or short sleep duration, with 7-8 hours of sleep being considered more appropriate (31). Follow-up studies of CKD patients have also shown that those who experience sleep deprivation or deteriorating sleep quality tend to experience a faster decline in eGFR and are more susceptible to progressing to ESKD (32). Additionally, a prospective and longitudinal study involving CKD patients demonstrated that depressive symptoms at baseline are linked to progression to ESRD, mortality, or hospitalization in CKD patients (33). In our study, we also found a longitudinal association between CircS and incident CKD, providing more evidence in this topic. However, this study was performed in the aging Chinese individuals, needing further verification in the overall population.

In our study, baseline survey results indicated that individuals in the CircS group tended to be older, female, divorced/separated/widowed, illiterate, less likely to smoke and drink alcohol, but had higher BMI, LDL, total cholesterol, CRP, uric acid, and hemoglobin levels. They also had a higher prevalence of depression, reduced sleep duration, and hypertension. It may be speculated that women experiencing menopause, shorter sleep duration, and depression are at a higher risk of CKD. However, surprisingly, subgroup analysis revealed an increased risk of prevalent CKD among married or cohabiting participants. This suggests that divorce, separation, or widowhood may play a protective role. One could hypothesize that these life changes might alleviate certain stressors such as family disputes, household responsibilities, or unhealthy relationships, while also prompting individuals to pay greater attention to their health and adopt healthier lifestyles. These factors may collectively contribute to a reduced risk of CKD. It’s important to emphasize that our study is observational in nature and cannot establish causation. Additionally, the relationship between these factors and CKD may be influenced by other underlying variables, necessitating further research for a deeper understanding of this association.

One fundamental assumption explaining these associations is the potential misalignment between external behavioral patterns and endogenous molecular circadian clocks. Kidneys possess peripheral circadian clocks that facilitate the self-sustained rhythmicity of the glomerular filtration rate (29). Studies have demonstrated that the mammalian circadian system operates as a hierarchical multi-oscillator structure (34). The central clock, situated in the SCN of the hypothalamus, orchestrates peripheral clocks distributed throughout the body (2). Given that light serves as the primary external cue for synchronizing the central circadian clock in the SCN (29), it is reasonable to hypothesize that chronic circadian disruption resulting from factors such as shift work, irregular sleep patterns, or artificial lighting may exert an influence on peripheral oscillators, potentially leading to a decrease in eGFR. Another plausible mechanism through which CircS may contribute to declining eGFR involves the presence of psychological and psychosocial stressors (35). Stress can induce renal vasoconstriction by stimulating the sympathetic nervous system, resulting in reduced RPF and GFR (29). Furthermore, persistent activation of the hypothalamic-pituitary-adrenal axis due to chronic external stressors associated with CircS can activate the sympathetic nervous system. Activation of the renal SNS, in turn, may impact renal function through the renin-angiotensin-aldosterone system (RAAS) (36). It has been observed that individuals with sleep disorders exhibit heightened daytime sympathetic nervous system activity (37) and fail to enhance cardiac vagal tone during the transition from wakefulness to non-rapid eye movement (38). Moreover, the overactivation of the RAAS not only leads to an increase in intra-glomerular pressure but also damages vascular endothelial cells, activates reactive oxygen species (ROS), and inhibits sympathetic hyperactivity and nitric oxide (NO), all of which are well-established risk factors for renal damage (39, 40).

Furthermore, canonical clock genes such as Clock, Bmal1, Rev-erbα, Cry1, Cry2, Per1, and Per2 exhibit cyclical expression and/or activity within cells, driving cell-autonomous circadian rhythms (8, 41, 42). Clock genes play a crucial regulatory role in the inflammatory response, and therefore (43), CircS may lead to systemic inflammation, which could result in endothelial dysfunction within glomeruli and subsequent renal impairment, thereby promoting CKD progression. For instance, the circadian clock protein BMAL1 regulates interleukin-1β (IL-1β) in macrophages through the nuclear factor erythroid 2-related factor 2 (Nrf2) pathway (44). Nrf2 expression is upregulated in the glomeruli of lupus nephritis (LN) patients, and in LN mouse models, Nrf2 mitigates LN by inhibiting oxidative injury and the NF-κB-mediated inflammatory response (45). Additionally, hypertension is a significant etiological factor for CKD development and progression (46), particularly with mean sleep systolic blood pressure recognized as one of the most important independent predictors of CKD (47). For every one standard deviation decrease in mean sleep systolic blood pressure, the risk of CKD decreases by 27% (48). Normal blood pressure exhibits a clear circadian rhythm, with elevated blood pressure upon awakening in the morning and a decrease during nocturnal sleep (41, 49). This rhythmicity is regulated by various factors with circadian patterns, including environmental cues, the nervous system, the hypothalamus-pituitary-adrenal axis, vascular factors, and the kidneys (49). Consequently, disruptions in circadian rhythms that lead to non-dipping nocturnal blood pressure (non-dipper hypertension) are associated with the occurrence and progression of CKD, although the underlying molecular mechanisms remain unclear (50, 51).

The limitations of this study include the lack of repeated measurements for CircS and some potential covariates. Additionally, the follow-up period was relatively short, and a 4-year follow-up may not be sufficient to detect more pronounced effects of CircS. All sleep information was collected through self-reports, which may be subject to recall bias and misclassification, rather than objective measurements of sleep parameters. Future research should focus on objective sleep monitoring methods, such as polysomnography (52), wrist actigraphy and sleep diaries (31). Non-alcoholic fatty liver disease (NAFLD) was initially proposed as a component of CircS (53). It is well-known that the prevalence of NAFLD in China is high (approximately 20%) (54). However, we did not have information about NAFLD in our study. Finally, because our study participants were Chinese, the generalizability of our findings may be limited by ethnic differences. Future research should examine whether our findings can be applied to other populations. Despite these limitations, our study had a large sample size, was nationally representative, had a high follow-up rate, and collected detailed data for each participant. Furthermore, we adjusted for a wide range of covariates to explore the independent influence of CircS on the incidence of CKD.

## 5 Conclusion

In summary, our study was conducted in a middle-aged and elderly Chinese population and provided evidence that CircS is a risk factor for CKD. However, the mechanisms by which CircS leads to CKD require further investigation.

## Data availability statement

The original contributions presented in the study are included in the article/supplementary material, further inquiries can be directed to the corresponding author/s.

## Ethics statement

The ethical review board of Peking University meticulously examined and subsequently sanctioned this study (IRB 00001052-11014). The studies were conducted in accordance with the local legislation and institutional requirements. The participants provided their written informed consent to participate in this study.

## Author contributions

YX: Writing – original draft, Visualization, Software, Methodology, Formal Analysis. QZ: Writing – original draft, Formal Analysis, Conceptualization. YZ: Writing – review & editing, Software, Project administration, Methodology, Formal Analysis. ZL: Writing – review & editing, Supervision, Project administration, Methodology, Conceptualization. XW: Writing – review & editing, Supervision, Project administration, Funding acquisition, Conceptualization.
